# The role of the brain renin-angiotensin system in Parkinson´s disease

**DOI:** 10.1186/s40035-024-00410-3

**Published:** 2024-04-15

**Authors:** Jose Luis Labandeira-Garcia, Carmen M. Labandeira, Maria J. Guerra, Ana I. Rodriguez-Perez

**Affiliations:** 1 Cellular and Molecular Neurobiology of Parkinson´S Disease, Research Center for Molecular Medicine and Chronic Diseases (CIMUS), IDIS, University of Santiago de Compostela, Santiago de Compostela, 15782 Spain; 2 Networking Research Center On Neurodegenerative Diseases (CIBERNED), Madrid, Spain; 3 Neurology Service, University Hospital of Ourense, Ourense, Spain

**Keywords:** Angiotensin, Dopamine, NADPH-oxidase, Neurodegeneration, Neuroprotection, Neuroinflammation, Oxidative stress, Parkinson

## Abstract

The renin-angiotensin system (RAS) was classically considered a circulating hormonal system that regulates blood pressure. However, different tissues and organs, including the brain, have a local paracrine RAS. Mutual regulation between the dopaminergic system and RAS has been observed in several tissues. Dysregulation of these interactions leads to renal and cardiovascular diseases, as well as progression of dopaminergic neuron degeneration in a major brain center of dopamine/angiotensin interaction such as the nigrostriatal system. A decrease in the dopaminergic function induces upregulation of the angiotensin type-1 (AT1) receptor activity, leading to recovery of dopamine levels. However, AT1 receptor overactivity in dopaminergic neurons and microglial cells upregulates the cellular NADPH-oxidase-superoxide axis and Ca^2+^ release, which mediate several key events in oxidative stress, neuroinflammation, and α-synuclein aggregation, involved in Parkinson's disease (PD) pathogenesis. An intraneuronal antioxidative/anti-inflammatory RAS counteracts the effects of the pro-oxidative AT1 receptor overactivity. Consistent with this, an imbalance in RAS activity towards the pro-oxidative/pro-inflammatory AT1 receptor axis has been observed in the substantia nigra and striatum of several animal models of high vulnerability to dopaminergic degeneration. Interestingly, autoantibodies against angiotensin-converting enzyme 2 and AT1 receptors are increased in PD models and PD patients and contribute to blood–brain barrier (BBB) dysregulation and nigrostriatal pro-inflammatory RAS upregulation. Therapeutic strategies addressed to the modulation of brain RAS, by AT1 receptor blockers (ARBs) and/or activation of the antioxidative axis (AT2, Mas receptors), may be neuroprotective for individuals with a high risk of developing PD or in prodromal stages of PD to reduce progression of the disease.

## Background

The renin-angiotensin system (RAS) was identified for the first time by Tigerstedt and Bergman (1898) [1] in the rabbit’s kidney. Phylogenetically, the RAS is a very old hormone system, present in primitive vertebrates such as the lamprey, playing a key role in the adaptation from aquatic to terrestrial life [2, 3] and the continuation of life in little salt ecosystems [4]. This was related to the major role of RAS, as a circulating hormonal system, in the regulation of blood pressure and sodium and water homeostasis, which was the classical function associated with RAS for decades. Then, local or paracrine RAS were observed in different tissues and organs, including the brain. In peripheral tissues, although both the circulating RAS and the local tissue RAS may act together, the circulating RAS appears less important than the local RAS in the RAS tissue effects [5].

Dopamine was synthesized in 1910 [6], and dopamine deficits in patients with Parkinson's disease (PD) were initially observed by Ehringer and Hornykiewicz (1960) [7]. Later, the role of dopamine in peripheral organs, particularly in the renal and cardiovascular systems, was identified [8]. More recently, different functions of dopamine in peripheral organs and tissues have been revealed, including regulation of blood pressure, sodium and water homeostasis, gut motility, respiration, and immune responses [9, 10]. In addition to brain cells, dopamine D1-like and D2-like receptor subtypes have been observed in peripheral tissues such as the blood vessels, kidney, heart, adrenal gland, gastrointestinal tract sympathetic nerve terminals, and almost all immune cell subpopulations [11, 12].

Interestingly, important functional interactions between the local dopaminergic and angiotensin systems have been observed in several peripheral organs. Dysregulation of the interactions between both systems, such as dysregulation of the expression or dimerization between dopamine receptors and angiotensin receptors, results in renal degenerative diseases and hypertension [13, 14]. Over the last decades, similar interactions between the dopaminergic system and the local RAS have been observed in the brain. Several studies from our research group and others have revealed a major role of the dysregulation of dopamine-RAS interactions in brain diseases, particularly in dopaminergic degeneration in PD, as detailed in the following sections.

**Fig. 1:**
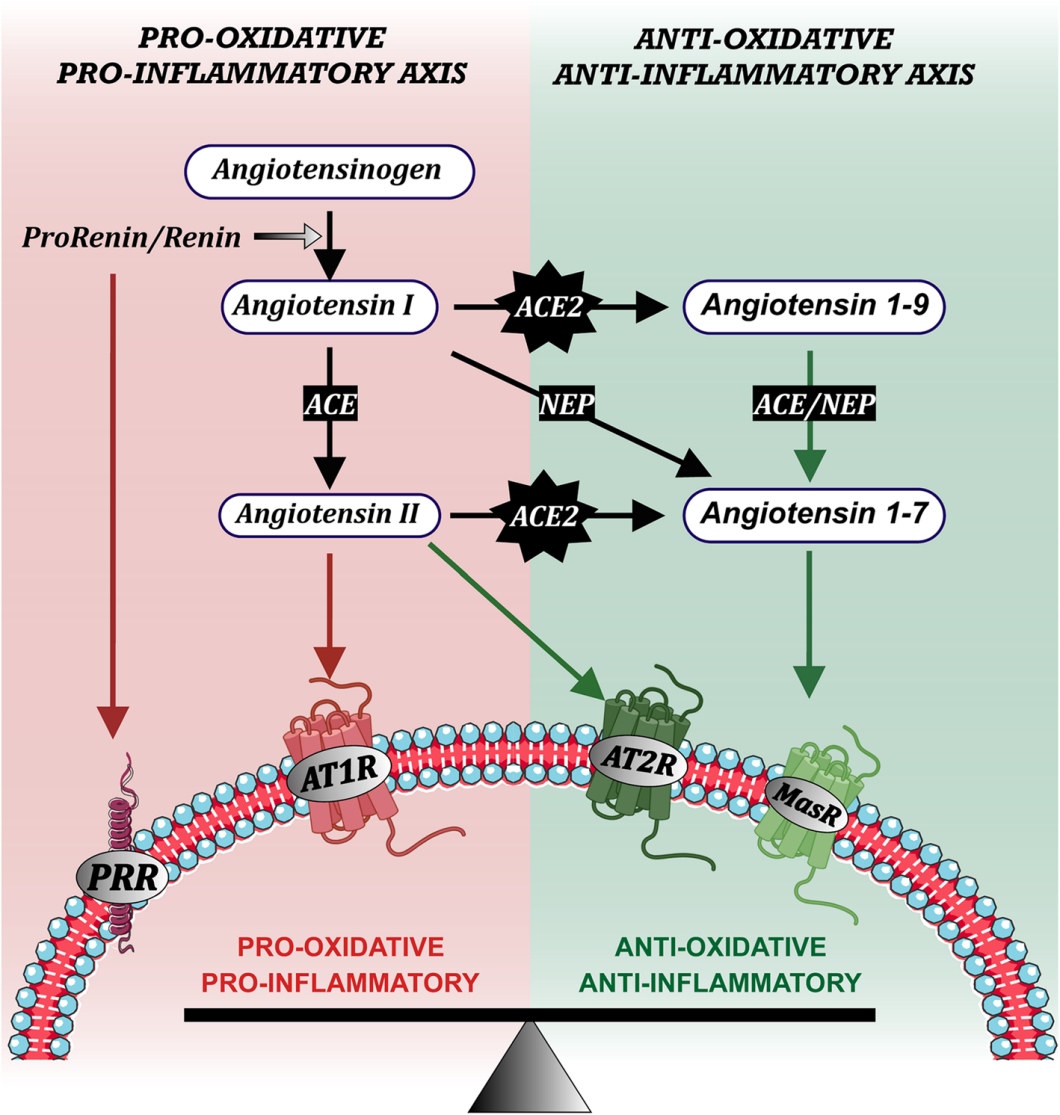
The renin-angiotensin system (RAS) is organized into two opposite arms that must be properly balanced: a pro-oxidative/pro-inflammatory axis (in red), mainly formed by Angiotensin II that binds AT1 receptors (AT1R), and an antioxidative/anti-inflammatory axis (in green), mainly formed by Angiotensin II-binding AT2 receptors and Angiotensin 1–7-binding Mas receptors (MasR) or Mas-related G protein-coupled receptors. The enzyme prorenin/renin acting on the precursor protein angiotensinogen produces Angiotensin I, which is converted to Angiotensin II by the angiotensin-converting enzyme (ACE or ACE1). Renin and its precursor prorenin (PR) can also bind specific pro-oxidative PR receptors (PRR). Angiotensin-converting enzyme 2 (ACE2; also known as the major entry receptor for the SARS-COV viruses) plays a major role in balancing both RAS arms, as ACE2 (together with other peptidases such as Neprilysin, NEP) transforms peptides of the pro-inflammatory axis (Angiotensin I and, particularly, Angiotensin II) into peptides of the anti-inflammatory axis (Angiotensin 1–9 and, particularly Angiotensin 1–7)

Angiotensin II (AngII) is the main RAS effector peptide, produced from the precursor protein angiotensinogen through sequential cleavages by the enzymes renin and angiotensin-converting enzyme (ACE, ACE1) (Fig. 1). AngII binds two major G-protein coupled receptors (GPCR) named AngII type 1 (AT1) and 2 (AT2) receptors. The AT1 receptors are related to most of the classical RAS peripheral effects, such as vasoconstriction and kidney water and salt retention. The human AT1 receptor gene is localized on chromosome 3q, coding for a 40–42 kDa protein (359 amino acids). AT1 receptor activation promotes hydrolysis of membrane phosphatidylinositol-4,5-bisphosphate, which produces inositol trisphosphate (IP3) and diacylglycerol (DAG). IP3 binds IP3 receptors, which are ligand-gated Ca^2+^ release channels located in intracellular Ca^2+^ store sites (such as the endoplasmic reticulum), inducing mobilization of intracellular Ca^2+^ stores [15, 16]. DAG activates protein kinase C, which promotes the activation of the NADPH oxidase complex [17, 18], the second major source of superoxide after mitochondria [19, 20]. NADPH-oxidase-derived superoxide and superoxide-derived reactive oxygen species (ROS) are major factors responsible for the pro-oxidative and pro-inflammatory effects of AT1 receptor activation [21, 22].

The AT2 receptor is a protein of 363 amino acids [23, 24]. The human AT2 gene is located in the X chromosome [25], particularly in the Xq23 region [26]. AT2 receptor effects are normally opposed to those induced by AT1 receptors. AT2 receptor activation decreases the NADPH oxidase activity and superoxide production, and inhibits NFκB and ERK1/2 phosphorylation, with nitric oxide being a key second messenger for AT2 signaling [27–29]. Sex differences in AT2 expression have been observed, which may be related to both hormonal and sex chromosome complement effects [30].

Beyond the classical AT1 and AT2 receptors, additional angiotensin peptides and receptors modulate the RAS function, which is overall organized into a pro-oxidative and pro-inflammatory arm and a protective or counter-regulatory anti-oxidative and anti-inflammatory arm (Fig. 1). Both arms must be correctly balanced in physiological conditions. The pro-oxidative/pro-inflammatory axis is mainly constituted by AngII/AT1 receptor activation, which upregulates the function of the NADPH-oxidase complex and induces Ca^2+^ release, as described above. In addition, renin and its precursor prorenin also act on the corresponding receptors (Fig. 1). Binding of prorenin to its receptor provides prorenin with catalytic properties similar to those of renin. Furthermore, the prorenin receptor induces a signaling pathway resulting in pro-oxidative effects as those induced by AT1 receptor activation [31]. The anti-oxidative/anti-inflammatory axis is mainly constituted by the AngII/AT2 receptor component, and by the activation of the G-protein-coupled receptor Mas [32, 33] and the Mas-related GPCR members (Mrg), such as MrgD [34] and MrgE [35] by Ang(1–7) and alamandine (Fig. 1). The angiotensin-converting enzyme 2 (ACE2) plays a key role in the balance between the two RAS arms because ACE2 converts peptides of the pro-oxidative arm (AngI and AngII) into components of the anti-oxidative arm such as Ang1-9 and, particularly, Ang1-7 [36–38]. Alamandine is generated by decarboxylation of the Asp residue of angiotensin-(1–7) [34]. The role of other RAS components such as AngA, AngIII, and AngIV is more controversial (see for review [34, 39]).

ACE2 has been intensely studied over the last few years because ACE2 is the primary binding site for SARS-CoV-2 entry into host cells [40, 41]. Many studies have suggested a major role of the tissue RAS in the pathophysiology and severity of COVID-19 [42, 43], as viral binding reduces the ACE2 levels at the cell surface [44], which leads to a shift in the RAS balance toward inflammation and disease severity. This raised the dilemma of increasing ACE2 levels in tissues to inhibit the inflammatory responses or decreasing tissue ACE2 levels to inhibit viral entry and replication. Similarly, the possible beneficial or detrimental effects of therapies with ARBs and ACE inhibitors were highly controversial [45, 46]. Research [47, 48] and clinical studies [49, 50] showed the non-detrimental and possible beneficial effects of these drugs. This has been detailed in our recent review article [51]. Interestingly, a possible increase in the risk of PD related to COVID-19 has been suggested [52, 53]; however, the possible involvement of RAS dysregulation in this link remains to be studied.

In addition to the classic circulating RAS and the tissue or paracrine RAS (Fig. 1), an intracellular or intracrine RAS has been described in several types of cells, including fibroblasts, vascular smooth muscle cells, cardiac cells, kidney cells, and neurons [38, 54, 55]. The existence of a third level of RAS is supported by the intracellular synthesis of AngII and the intracellular location of different RAS components and receptors. Although the role of the intracellular RAS is still unclear, our data in neurons suggest cell protective effects, as described below [35, 38, 56–58].

The complexity of the RAS functioning is further increased by the possible formation of receptor complexes. Over the last decades, research on GPCRs revealed that individual receptors can interact to form heteroreceptor complexes, leading to new functional units providing cell responses that may differ from those of the individually acting receptors [59]. Regarding RAS GPCRs, receptor heteromers have been observed both between different RAS receptors and between RAS receptors and receptors of different systems such as dopaminergic, adenosine, cannabinoid, bradykinin, and β-adrenergic receptors [60–62]. The AT1 receptor dimerizes with itself and forms AT1-AT1 homodimers [63]. AT1-AT2 receptor association leads to AT1 signal inhibition [64], and administration of an antagonist of one receptor releases the inhibition of the partner receptor activity [65]. In AT1-CB1 heteromers, the CB1 receptor can modulate the AT1-mediated signaling [66]. We have also observed that Mas receptor can interact with the AT1 receptor and/or AT2 receptor to form heterodimers and heterotrimers in microglia and neurons [61]. In the striatum, we observed that the AT1 receptor forms heteromers with dopamine D2 receptors and that AT1 agonist and antagonist drugs can selectively alter the functional responses of the D2 receptors [67].

### The existence of a local brain RAS. RAS in the dopaminergic nigrostriatal system

In the brain, the RAS was initially associated with blood pressure regulation through the circumventricular organs [68], as AngII cannot cross a healthy BBB [69]. However, brain levels of AngII are higher than the circulating levels [70], suggesting the presence of a brain paracrine RAS. Astrocytes are the major source of the precursor protein (angiotensinogen) for the brain RAS paracrine system [70–72], with minor contributions from other cells such as neurons [38, 73, 74]. Initially, some authors were unable to detect renin in the brain and suggested that the brain AngII may be uptaken from circulating AngII, thus questioning an independent brain RAS [75]. Different studies detected low levels of renin [76, 77] and, essentially, high levels of prorenin and prorenin receptors in the brain. Prorenin activation by the prorenin receptors confers a catalytic function of prorenin like that of renin [31, 78, 79]. In addition, more recent studies suggest that, although angiotensin peptides cannot cross a healthy BBB, pathological upregulation of peripheral RAS components may modify the BBB integrity [80, 81].

Initially, several studies detected the presence of RAS components in the basal ganglia, particularly in the nigrostriatal system [82–84]. More recently, a paracrine local RAS was observed in the substantia nigra (SN) and striatum of rodents [22, 79, 85], as well as non-human primates and humans [86, 87]. In dopaminergic neurons and glial cells, the presence and functional effects of different components of the pro-oxidative/pro-inflammatory arm have been shown, including the AngII/AT1 receptor axis [22, 85, 88] and the pro-renin signaling pathway [78, 79]. The presence and functional effects of the anti-oxidative/anti-inflammatory arm have also been shown, including the AngII/AT2 axis [30, 89] and the Ang1-7/ Mas receptor pathway [56]. Consistent with this, a recent study using single‐cell genomic profiling of human dopaminergic neurons revealed high expression of the AT1 receptor gene as a marker of most vulnerable dopaminergic neurons in humans, including PD patients, which are located in the ventral tier of the SN pars compacta (SNpc) [90], further supporting the potential role of the pro-oxidative AngII/AT1 receptor axis in dopaminergic degeneration, as detailed below.

### Angiotensin-dopamine interactions in the nigrostriatal system

Dopamine is involved in cardiovascular, renal, endocrine, gastrointestinal, and immune functions [11, 12, 91–93], and different dopamine D1-like and D2-like receptors are located in peripheral tissues [11, 12]. In these tissues, a functional interaction between the local RAS and dopamine has been shown. This has been particularly studied in kidney regulation of sodium and water homeostasis and cardiovascular regulation, observing that the dopaminergic system and RAS counter-regulate each other [94, 95]. Furthermore, dimerization between receptors of both systems has been observed in peripheral cells [60]. Interestingly, dysregulation of interactions between the two systems, including the imbalance between dopamine and angiotensin receptor expression [13] or alterations in dopamine or angiotensin levels [14], leads to pathological processes such as renal degenerative diseases and hypertension.

In the brain, an interaction between dopamine and AngII was initially shown by microdialysis, which revealed that acute striatal administration of AngII induced a release of dopamine in the striatum that could be blocked by AT1 receptor antagonists [96, 97]. These results suggest that AngII, by activating AT1 receptors, induces dopamine release and that a decrease in dopamine levels may induce a compensatory increase in AngII/AT1 receptor activity to restore the dopaminergic function. AngII has also been shown to modulate the expression and trafficking of key enzymes for catecholamine biosynthesis such as tyrosine hydroxylase and dopamine β-hydroxylase, thus regulating the synthesis of norepinephrine and dopamine [98]. Processes of counter-regulation between angiotensin and dopamine receptors have been shown in the SN and striatum in several studies [99, 100]. Dopamine depletion, in 6-hydroxydopamine (6-OHDA) or reserpine models, upregulates the AT1/NADPH-oxidase activity, which can be reversed after restoring dopamine levels by L-DOPA administration [100]. Furthermore, both D1 and D2 KO mice showed upregulation of AT1 receptor expression [99]. Dimerization between receptors of the two systems has also been observed in the nigrostriatal system [61, 67]. As AT1 activation induces dopamine release, and D2 autoreceptors in dopaminergic neurons modulate dopamine release, the formation of AT1/D2 heteromers appears particularly interesting. It is also interesting to note that the striatal dopamine depletion (e.g., using 6-OHDA) and treatment with L-dopa modify the levels of different types of RAS-receptor heteromers [61, 101].

### Dysregulation of RAS-dopamine interactions and dopaminergic degeneration

**Fig. 2:**
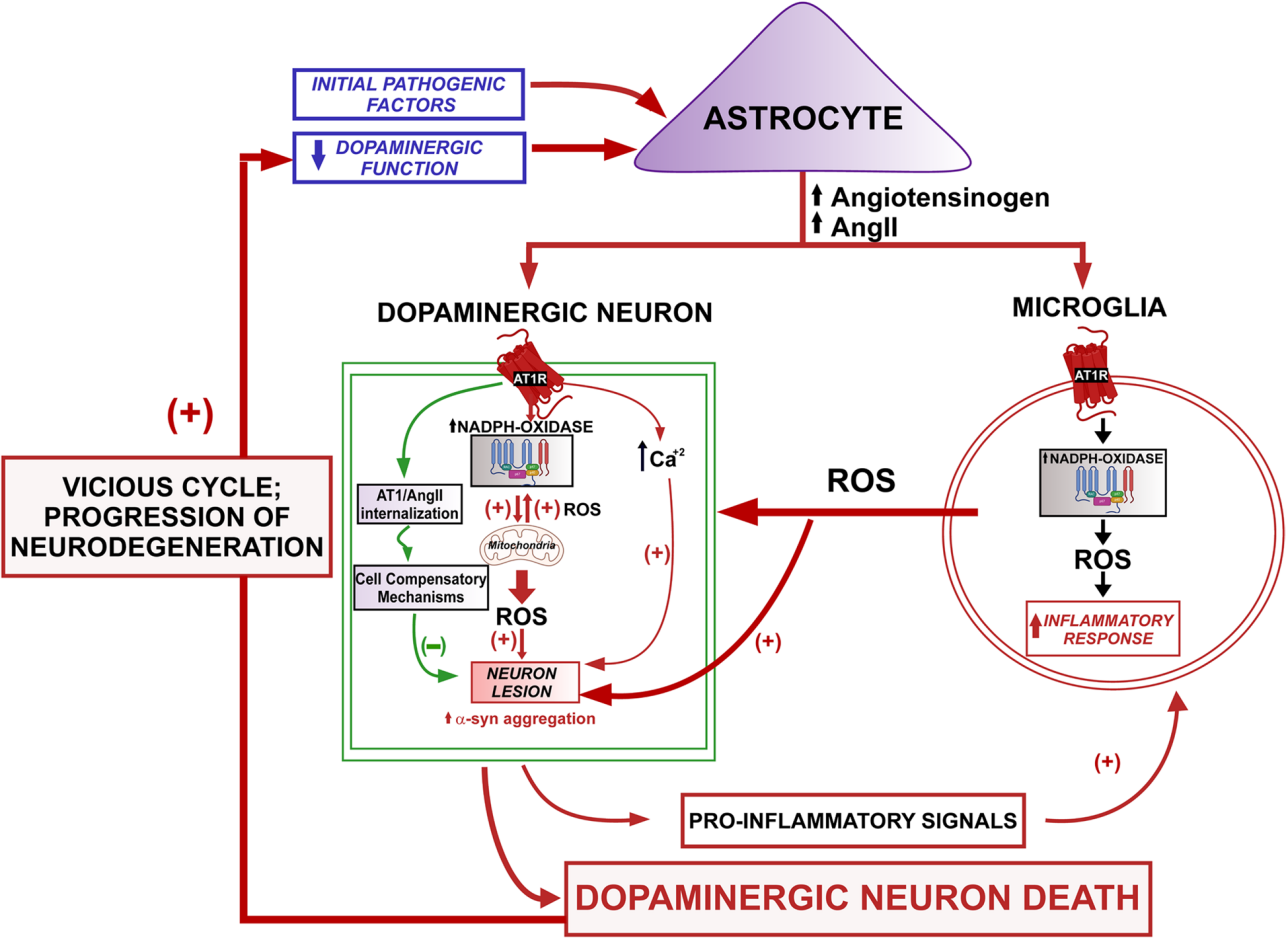
The brain RAS plays a role in the progression of dopaminergic neuron degeneration. Different pathogenic factors may trigger molecular and cellular changes that lead to an initial dysregulation of the brain RAS, or dysregulation of the dopaminergic neuron function leading to decreased dopamine production, which affects the dopamine/RAS interaction in neurons and glial cells. In neurons, a decrease in dopamine level upregulates the angiotensin type-1 (AT1) receptor activity, leading to the recovery of dopamine levels together with overactivation of the NADPH-oxidase-superoxide-mitochondria axis and Ca^2+^ release, which mediate several key events such as oxidative stress, α-synuclein aggregation, and neuroinflammation involved in the progression of Parkinson's disease (PD). An intraneuronal antioxidative/anti-inflammatory RAS counteracts the effects of the pro-oxidative AT1 receptor overactivation (detailed in Fig. 3). In microglial cells, AT1 receptor upregulation activates the NADPH-oxidase complex, increasing the release of ROS to the extracellular space and the inflammatory response. In astrocytes, a decrease in dopamine level induces an increase in paracrine angiotensinogen/AngII production that can act on neurons and microglial cells. AngII, angiotensin II; AT1, angiotensin type I; ROS, reactive oxygen species

As previously reported in cardiovascular and renal tissues, dysregulation of the RAS/dopamine interactions in the nigrostriatal system promotes neuroinflammation and dopaminergic neurodegeneration [99, 102] (Fig. 2). The possible deleterious effect of RAS/dopamine dysregulation has been studied in several in vivo and in vitro models of PD. In animal models, the dopaminergic lesion induces a loss of dopamine and upregulation of the pro-oxidative/pro-inflammatory AngII/AT1 receptor axis, enhancing the progression of dopaminergic degeneration. This has been observed in our studies and reports from different laboratories using the main in vivo PD models including neurotoxic models such as the 6-OHDA [22, 103] and the MPTP models [21, 85, 104, 105] and, more recently, PD models based on over-expression of α-synuclein using adeno-associated viral vectors [106]. The dopaminergic lesion is significantly decreased by ACE inhibitors [104, 107, 108] and, more specifically, by the blockage of AT1 receptors [22, 85, 103, 106]. In vitro models, such as primary mesencephalic cultures treated with low doses of the neurotoxin 6-OHDA or MPP^+^ revealed similar results, showing that the neurotoxin-induced dopaminergic neuron death is significantly increased by administration of AngII and inhibited by treatment with AT1 receptor blockers [22, 85].

Over the last decade, a series of clinical observations also supported that the overactivation of the AngII/AT1 pro-inflammatory axis contributes to the progression of PD. Early studies reported increased ACE activity in the cerebrospinal fluid (CSF) of PD patients [109], associations between ACE-genetic polymorphisms and PD [110], and beneficial effects of ACE inhibitors in PD patients [111]. The effects of antihypertensive drugs on the risk of PD have been studied in several cohort studies [112, 113]. However, the conclusive value of these studies was limited by the low number of patients or other confounding factors such as short periods of exposure to the analyzed drug or the inclusion of different anti-hypertensive drugs or different ARBs and ACE inhibitors in the same group of patients [114]. More recent studies also suggest the potential clinical effects of ARBs [115, 116], including studies using artificial intelligence [117], which support the neuroprotective effects of AT1 receptor blockers on PD risk. Recent retrospective cohort studies involving a large number of patients are particularly interesting and show that ARBs may be an effective neuroprotective strategy to reduce PD risk and progression [118, 119]. ARB treatment is associated with a marked reduction of PD risk in patients with recently diagnosed hypertension [119]. Furthermore, ARBs with BBB-penetrating properties and a high cumulative duration of treatment are particularly effective [118].

The development of prodromal clinical trials, using ARBs that can cross the BBB or other molecules able to inhibit the effects of AT1 receptor over-activity, is further supported by recent key data from Kamath et al. [90]. Using single-nucleus RNA sequencing and unbiased clustering analysis of human SN dopaminergic neurons, they identified one neuron population showing high levels of *SOX6* and AT1 receptor gene (*AGTR1*), specifically localized in the most vulnerable region (i.e., the ventral tier) of the SNpc. Consistent with this, the highest loss of neurons in PD patients and patients with Lewy body dementia relative to controls was observed in the *SOX6*_*AGTR1* subpopulation, and the levels of *AGTR1* expression correlated with the susceptibility to neurodegeneration in neurons. Similar results were found in the SNpc of other mammalian species [90, 120]. Interestingly, compared to more resistant neurons in other regions of the SN and other brain regions, these most vulnerable neurons in the ventral tier of the SNpc also show lower levels of buffer mechanisms, such as low calbindin levels, to counteract rising intracellular calcium [121, 122]. As described above, the major consequences of AT1 receptor overactivation are the NADPH-oxidase-derived oxidative stress and intracellular calcium mobilization (Fig. 2).

### Mechanisms of the increase in dopaminergic degeneration induced by brain RAS dysregulation

In both peripheral tissues and the brain, NADPH-oxidase-derived oxidative stress, intracellular calcium dysregulation, and enhanced inflammatory responses appear to mediate the deleterious effects of overactivation of the pro-oxidative/pro-inflammatory axis of the tissue RAS (Fig. 2; Table 1). It is known that oxidative stress and neuroinflammation are early components of dopaminergic degeneration, and both factors, probably acting synergistically with additional factors, lead to the progression of PD [123–125]. Consistent with the role of NADPH-oxidase activation and NADPH-oxidase-derived ROS in the exacerbation of dopaminergic neurodegeneration induced by AT1 receptor overactivation, neurodegeneration is inhibited by blockers of NADPH-oxidase activation in animal models [22, 85, 103]. Furthermore, both angiotensin receptors and NADPH-oxidase components have been observed in dopaminergic neurons and glial cells in the SN of different mammals, including humans [22, 85, 87]. As previously observed in peripheral processes, particularly in kidney and cardiovascular tissues, AngII acts both on tissue-resident cells (neurons in the brain) and inflammatory cells (glia, particularly microglial cells in the brain) (Fig. 2). Activation of AT1 receptors in dopaminergic neurons induces upregulation of NADPH-oxidase-derived superoxide and intracellular ROS levels (Fig. 2). As observed in other types of cells [126, 127], the NADPH oxidase-derived ROS interact with the mitochondria in dopaminergic neurons [105, 128, 129], via mitochondrial ATP-sensitive potassium channels, leading to a further increase in mitochondrial ROS production; inversely, the mitochondrial-derived ROS further increase NADPH-oxidase activation and ROS generation, leading to a vicious cycle [20, 105]. In addition, the NADPH-oxidase-derived ROS can act as a second messenger in cellular signaling pathways, including those triggering the inflammatory response and the migration of inflammatory glial cells into the affected region [19, 20, 130]. Furthermore, we have already indicated that AT1 receptor activation, via IP3, induces intracellular calcium mobilization. Therefore, overactivation of AT1 receptors can induce dysregulation of cell calcium homeostasis, which has been involved in PD pathogenesis, as physiological calcium levels must be correctly balanced to prevent neuronal death [131–133].

**Table 1:** Dopaminergic degeneration induced by RAS dysregulation (AngII/AT1 axis overactivity)

**Mechanisms of AT1-induced neuron degeneration**	**References**	**Possible processes triggering AT1 overactivity**	**References**
Neuronal NADPH-oxidase overactivity (↑ Oxidative stress)	[17, 20, 22, 85, 87, 105, 127–129]	Decline in dopaminergic function	[99, 100, 102, 134–138]
Neuronal mitochondrial dysfunction (↑ Oxidative stress)	[35, 38, 56–58, 127–129, 139]	Aging-related processes	[35, 56, 57, 89, 99, 140–149]
Neuronal calcium dysregulation	[15, 16, 121, 122, 132, 133]	Menopause	[150–155]
Increase in α-synuclein aggregation	[156]	Chronic brain hypoperfusion	[157–159]
Increase in α-synuclein transmission	[156]	RAS-related autoimmunity	[80, 81]
Upregulation of the microglial inflammatory response	[20, 22, 30, 78, 88, 89, 140, 160–164]	Metabolic syndrome	[81, 165]
		Gastrointestinal processes	[91–93, 166–169]
		Microbiota dysregulation	[170–176]

RAS receptors were also observed in glial cells (Fig. 2), including microglia, indicating that AngII can act directly on their receptors to modulate the neuroinflammatory response. In inflammatory cells, such as microglia, NADPH oxidase activation can induce high levels of ROS that are released to the extracellular space, exacerbating oxidative stress in neurons. In microglia, the activation of NADPH-oxidase also induces low levels of intracellular ROS to act as a second messenger to modulate the inflammatory response [19, 20, 160]. Over the last decade, the major role of the RAS in the modulation of microglial inflammatory response has been shown in a considerable number of studies. In microglia, activation of the RhoA/ROCK pathway [88, 161, 162], release of TNF-α [163], and altered iron homeostasis [164] are involved in the enhancement of microglial response and dopaminergic degeneration by AngII/AT1 receptor activation (Fig. 2; Table 1). Up-regulation of NLRP3 inflammasome is also mediated by AT1 receptor activation [140]. Other RAS components also modulate the microglial response, such as AT2 receptors that promote the expression of anti-inflammatory responses [30, 89], and prorenin receptors that enhance inflammation and its damaging effects on dopaminergic neurons [78].

In addition to oxidative stress and neuroinflammation, a major factor involved in PD progression is α-synuclein aggregation and cell-to-cell transmission. However, it was unknown whether AngII/AT1 receptor overactivation affects α-synuclein aggregation and transmission in neurons and glial cells. In recent experiments [156], we observed that AngII/AT1 receptor overactivation promotes α-synuclein expression, aggregation, and glial transmission (Fig. 2; Table 1). This further supports the role of local RAS dysregulation in PD progression, and suggests that AT1 receptor blockers or RAS modulation, by enhancing the activity of the counterregulatory RAS receptors such as AT2 or Ang1-7/Mas receptors, is a promising therapeutic target for PD.

### Potential mechanisms and processes triggering the angiotensin/dopamine dysregulation

#### Functional decline of the dopaminergic system induces RAS dysregulation

Several major processes such as oxidative stress, neuroinflammation, and accumulation of α-synuclein aggregates are involved in the dopaminergic neurodegeneration in PD. However, their order of appearance is not clear and it remains unknown which of them acts first. It is frequently suggested that α-synuclein aggregation and Lewy body pathology are the initial processes triggering neurodegeneration. However, molecular and cellular changes originating from different genetic and/or environmental triggers may occur before α-synuclein aggregation, particularly affecting the most vulnerable neurons [177, 178]. An initial impairment in dopamine metabolism [179], a decrease in tyrosine hydroxylase [180], oxidative damage [181], and early neuroinflammation [182] have been suggested as initial triggers of the disease. Dysregulation of the brain RAS, and particularly, over-activation of the AngII/AT1 receptor axis are involved in all these potential triggering processes, and may be an early mechanism mediating the progression of dopaminergic neuron degeneration. However, the next question could be what the cause for the RAS dysregulation is (Table 1).

First, an initial decrease in dopamine levels (Fig. 2), due to dopamine homeostasis dysregulation, may lead to a compensatory increase in AngII/AT1 receptor activity to normalize dopamine levels. This may occur in the very initial stages of PD, aging, and other situations that have been associated with an increased risk of developing PD (see below). AngII/AT1 receptor overactivity may lead to a transitory upregulation of dopamine levels [67, 99, 100, 102, 134]. However, the AngII/AT1 receptor axis upregulation may simultaneously promote oxidative stress, neuroinflammation, and α-synuclein aggregation through increased NADPH-oxidase activity and alteration of intracellular calcium homeostasis. Furthermore, it is well known that dopamine is an immunomodulatory molecule, that dopamine receptors are present in immune effector cells [135, 136], and that dopamine inhibits NLRP3 inflammasome via dopamine receptors in several cell types [137, 138]. Therefore, an initial decrease in dopamine or dopaminergic function may enhance the inflammatory response, and upregulation of the pro-inflammatory RAS axis appears to be involved in this process (Fig. 2). Consistent with this, we have recently shown that dopamine regulates RAS activity in glial cells, modulating AngII release by astrocytes and the expression of angiotensin receptors in microglia [102]. Dopaminergic neurotoxins such as MPP^+^ can increase the release of angiotensinogen and AngII from astrocytes. However, dopamine, via type-2 (D2) receptors, down-regulates the production of angiotensinogen and the expression of AT1 receptors while increasing the expression of AT2 receptors in astrocytes. In microglia, dopamine administration induces downregulation of the AT1/AT2 ratio and inhibits the inflammatory response [102]. Furthermore, recent studies showed that AT1 receptors mediate microglial inflammasome complex activation in dopamine-depleted models [140].

Consistent with the role of RAS dysregulation in PD progression, overactivity of the pro-oxidative/pro-inflammatory AngII/AT1 receptor axis has been observed in several processes described below, which are known to increase the risk of PD.

#### Aging-related RAS dysregulation and PD

Different studies have shown that aging increases the vulnerability of dopaminergic neurons to degeneration. Advanced age is the first risk factor for PD and other neurodegenerative diseases. It has been shown that the pro-inflammatory and pro-oxidative state associated with aging (inflammaging) [183, 184] may favor the development of degenerative diseases [185, 186]. As RAS is involved in the neuroinflammatory response, the AngII/AT1 receptor upregulation in the nigrostriatal system may be a factor for increased dopaminergic vulnerability with aging. In aged male rats (see below for aged females), we observed higher levels of neuroinflammatory and oxidative markers in the SN and striatum, and an increase in the dopaminergic neuron death triggered by dopaminergic neurotoxins, which are downregulated by the AT1 receptor antagonist candesartan [99, 141]. AT1 receptor inhibition also ameliorates the upregulation of the NLRP3 inflammasome observed in aged rats [140, 187]. Aging-related upregulation of the pro-inflammatory axis components such as AT1 receptors and prorenin receptors has also been observed [89, 99, 142]. In dopaminergic neurons, this may be mediated by an age-related decrease in dopaminergic activity and the already mentioned RAS compensatory changes. Consistent with this, several studies have shown a loss of striatal D2 and D1 receptors in aged animals and humans [143, 144], and that the dopaminergic system is altered during normal aging [145, 146]. However, other non-dopaminergic factors appear also involved, as the age-related upregulation of the pro-oxidative/pro-inflammatory RAS has also been observed in other tissues apparently unrelated with the dopaminergic systems [147–149]. Interestingly, aged animals also show a decrease in the expression of components of the RAS anti-inflammatory axis, such as AT2 or Mas receptor expression, not only at the level of the cell membrane but also in the intracellular RAS system detailed below [35, 56, 57], revealing a decrease in the compensatory response of the RAS anti-inflammatory axis that contributes to the aging-related RAS imbalance.

#### Menopause, RAS dysregulation and PD

Menopause is also a risk factor for PD, as the incidence and the prevalence of the disease are higher in men and postmenopausal women than in premenopausal women of similar age [188, 189]. Over the last few decades, different experimental studies have reported the beneficial effects of estrogen against dopaminergic neurodegeneration [190, 191], and shown that the anti-inflammatory effects of estrogen are responsible for the neuroprotective effects [192, 193]. The beneficial effects of estrogen replacement therapies are more controversial [194, 195], with age and the period without estrogen before receiving the replacement treatment as underlying factors for the discrepancies. As estrogen-induced regulation of the RAS has been suggested to mediate the beneficial effects of estrogen in several peripheral tissues [150, 151], we studied the effects of the lack of estrogen on the nigrostriatal RAS in female rats with early surgical menopause (young ovariectomized rats) and natural menopause (aged rats) [152–155]. The activity of the RAS pro-oxidative/pro-inflammatory arm was increased in both groups of menopausal rats. Interestingly, treatment with the AT1 receptor blocker candesartan reduced the loss of neurons induced by low doses of dopaminergic neurotoxins in both groups of menopausal rats; however, the neuroprotection of the replacement therapy was effective only in the young rat groups [154]. We observed that there is a critical period for the neuroprotection with estrogen against dopaminergic neurodegeneration and that the local RAS plays a major role, as treatment with the AT1 receptor antagonist candesartan provided significant neuroprotection beyond the critical period for estrogen, [152]. The brain RAS system may be an efficient therapeutic target for the treatment or prevention of PD in estrogen-deficient women, together with or substituting estrogen replacement therapies [152].

Interestingly, recent studies have shown that levels of estrogen are not the only factor responsible for the reduced activity of the RAS pro-inflammatory axis in the nigrostriatal system of females relative to males and that the expression of the anti-inflammatory AT2 receptors is higher in females, independently of circulating levels of estrogen and probably related with the sex chromosome complement effects, which contribute to attenuate the inflammatory response [30].

#### Chronic brain hypoperfusion, RAS dysregulation and PD

Chronic brain hypoperfusion has also been related to an increased risk of neurodegeneration and PD [157, 158, 196]. This is consistent with previous studies in animal models showing that brain hypoperfusion enhances the neuronal loss induced by dopaminergic neurotoxins, together with the nigrostriatal expression of inflammatory markers such as IL-1β and increased oxidative stress such as increased NADPH-oxidase activity [159]. Interestingly, the hypoperfusion-induced changes are accompanied by upregulation of the AngII/AT1 receptor activity in the SN, and these changes are downregulated by chronic treatment with the AT1 receptor blocker candesartan [159].

#### RAS-related autoimmunity and PD

Autoimmune processes have also been involved in the triggering and progression of degenerative diseases, including PD [197–199]. We have recently observed an increase in the serum levels of autoantibodies for AT1 receptors (AT1 receptor agonistic autoantibodies, AT1-AA) and ACE2 autoantibodies (ACE2 antagonists) in PD patients compared to non-PD controls. We also found both autoantibodies in the CSF samples from some PD patients [80]. Furthermore, there was a significant correlation between serum levels of AT1-AA and serum inflammatory cytokines in PD patients but not in controls. In parallel experiments, using in vivo and in vitro PD models, we confirmed that these autoantibodies are associated with the neurodegenerative process and the accompanying neuroinflammatory changes, and led to further progression of neurodegeneration by increasing the activity of AT1 receptors and decreasing the activity of ACE2-related anti-inflammatory RAS axis, respectively. Consistent with the findings in PD patients, we observed a significant increase of these RAS-related autoantibodies in both serum and CSF of rats lesioned with the dopaminergic neurotoxin 6-OHDA. Furthermore, we confirmed in cultures that administration of AT1-AA increased the loss of dopaminergic neurons, which was inhibited by treatment with the AT1 receptor blocker candesartan [80]. Altogether, these findings suggest that the generation of RAS autoantibodies during early degenerative stages contributes to RAS dysregulation towards the proinflammatory axis and to the increased progression of dopaminergic degeneration and PD. ARBs or treatments that inhibit the generation of these autoantibodies may inhibit these effects.

#### Metabolic syndrome (MetS), RAS dysregulation and PD

In addition to the already mentioned risk factors, several peripheral diseases related to chronic inflammation, appear to increase the risk of neurodegenerative diseases including PD [200, 201]. MetS has been associated with chronic peripheral inflammation and increased risk of PD [202, 203]. As in the case of neurodegenerative diseases, MetS can be currently considered a silent epidemic disease. The definition of MetS consists of the presence of obesity and at least 2 of the following conditions: hypertension, hypertriglyceridemia, low HDL cholesterolemia, and type-2 diabetes/hyperglycemia [204]. In a rat model, we showed that MetS leads to the upregulation of the pro-oxidative/pro-inflammatory RAS axis in the SN, together with increases in oxidative stress, neuroinflammatory markers, and dopaminergic neurodegeneration, and all these changes were decreased by treatment with ARBs [81]. In rats, MetS also increases the circulating levels of major pro-inflammatory cytokines and 27-hydroxycholesterol. Interestingly, serum levels of pro-inflammatory AT1 and ACE2 autoantibodies are increased and correlated with several MetS parameters. AT1 and ACE2 autoantibodies are also present in the CSF of these rats. Osmotic minipump infusions of AT1 receptor autoantibodies disrupt BBB and affect the brain, leading to upregulation of the pro-inflammatory RAS activity in the SN and a significant increase in dopaminergic neurodegeneration in two different rat PD models [81]. Activation of AT1 endothelial receptors by the circulating agonistic AT1 receptor autoantibodies appears as a major mechanism of BBB disruption. This is consistent with several previous studies showing that stimulation of AT1 receptors in endothelial cells and perivascular macrophages by circulating AngII, but not hypertension itself, is a major mechanism of the BBB disruption observed in hypertension. Consistently, the disruption can be blocked by AT1 receptor blockers (ARBs) and not by other anti-hypertensive drugs [205–209]. In MetS patients, previous studies have also observed increases in the levels of pro-inflammatory cytokines [210, 211] and BBB permeability [212, 213], which were attributed to the increase in circulating cytokines. However, the increase in circulating agonistic AT1 receptor autoantibodies may also play a major role in BBB disruption, as patients with MetS show significantly higher levels of AT1 receptor autoantibodies, which lead to dysregulation of the SN RAS as observed in the MetS rat model [81]. In addition, circulating levels of AT1 receptor autoantibodies are significantly higher in non-Parkinsonian patients with MetS than in non-Parkinsonian patients without MetS. However, no significant difference has been observed between Parkinsonian patients with and without MetS, both showing higher levels of AT1 receptor autoantibodies than normal controls. This may be because dopaminergic degeneration and neuroinflammation in PD patients (without MetS) also lead to an increase in circulating autoantibodies [80], as detailed above. In MetS patients, both processes may trigger a vicious cycle that accelerates PD progression, which may be blocked by strategies against generation of these autoantibodies or by AT1 receptor blockers.

Additional mechanisms may be involved in the association between MetS and PD. Recent results from rat models of MetS suggest involvement of circulating extracellular vesicles (EVs) and their RAS cargo in the link between MetS and PD [81]. EV cargo shows the molecular state of their cells of origin [214], including the cellular level of RAS components [215]. Circulating EVs can cross the BBB and serve as inflammatory mediators [216, 217] and carriers of oxidative stress signals [218]. In the adipose tissues from obese animals, increases of angiotensinogen [219, 220], AT1 receptors [221],and prorenin receptors [222] have been reported. Consistent with this, we have recently observed that in rat models of MetS, EVs are highly increased in the serum and show increased pro-oxidative/pro-inflammatory and decreased anti-oxidative/anti-inflammatory RAS components, as well as increased inflammatory and oxidative stress markers. Interestingly, the increase of serum EVs, the RAS dysregulation and the increases of inflammatory and oxidative stress markers in the EV cargo are inhibited by chronical treatment with theAT1 blocker candesartan in MetS rats [165]. In vitro, administration of EVs isolated from the serum of MetS rats increases dopaminergic cell death and regulates the astrocytic function, leading to the upregulation of neuroinflammation and oxidative stress markers. The effects of treatment with EVs are inhibited by pre-treatment of cultures with the AT1 blocker candesartan [165]. Altogether, in MetS, circulating EVs may contribute, via RAS dysregulation, to the progression of neuroinflammation and dopaminergic cell death. This mechanism can be inhibited by treatment with ARBs such as candesartan.

#### Gastrointestinal processes, RAS dysregulation and PD

Many recent studies have suggested the association of gastrointestinal diseases with a higher risk of PD, and revealed the presence of a gut-brain axis. Gut dysmotility is a PD component, although the mechanisms are still unclear. Conversely, the role of gut diseases in PD remains to be clarified. Braak´s hypothesis suggested that PD may be caused by pathogens that act on the gastrointestinal tract, which induce gastrointestinal inflammation and oxidative stress, leading to α-synuclein deposition that is retrogradely transported to the brain [223, 224]. However, the gut-brain axis also regulates the nigrostriatal dopamine homeostasis, via the vagus nerve, as a caloric intake regulatory system [225, 226]. Consistent with this, a decrease in nigrostriatal dopamine level leads to changes in colonic expression of dopamine receptors as well as dopamine and acetylcholine levels in rodents, and experimental gut inflammation leads to changes in the nigrostriatal dopaminergic homeostasis [92]. Those results suggest that the nigrostriatal dopaminergic system and the gastrointestinal system interact bidirectionally and that both brain dopaminergic lesions and gastrointestinal lesions can lead to dysregulation of the functional interaction. This may explain the gastrointestinal alterations observed in PD patients, and the higher vulnerability of central dopaminergic neurons after gastrointestinal inflammation [92]. Several recent studies in rodents and humans support this proposition [227–229], and brain-first and body-first subtypes of PD have been proposed [228].

Interestingly, the gastrointestinal tract has a local RAS that is involved in major functional processes such as motility, absorption, and gastrointestinal inflammation [166–168]. The gastrointestinal RAS also plays a role in gut diseases such as inflammatory bowel disease and gastrointestinal motility disorders [167, 168]. As in the case of the nigrostriatal dopaminergic system, mutual regulation between the gastrointestinal dopaminergic and the angiotensin systems has been observed, which may be dysregulated with aging and by different processes, leading to increased vulnerability to gastrointestinal inflammatory diseases [91, 93]. Furthermore, nigrostriatal dopaminergic depletion also leads to upregulation of the pro-inflammatory axis of the gastrointestinal RAS, together with increased gut levels of oxidative stress and pro-inflammatory markers [92]. Conversely, experimental gastrointestinal inflammation leads to changes in dopaminergic homeostasis and upregulation of the pro-inflammatory RAS in the SN, which may contribute to increases in neuroinflammation, dopaminergic neuron vulnerability, and progression towards PD [92, 169].

#### Microbiota, RAS dysregulation, and PD

Recent studies suggest a connection of gut microbiota with neuroinflammatory and neurodegenerative disorders such as Alzheimer's disease and PD, and that intervention on microbiota may provide a novel strategy for treating and preventing neurodegeneration [230]. Although the exact mechanism remains to be clarified, gut microbiota and its metabolites may contribute to PD pathophysiology through modulation of gut inflammation (see above). However, additional mechanisms including the release of short-chain fatty acids or compounds affecting the BBB may also be involved [230].

Interestingly, the RAS, including systemic and gastrointestinal RAS, has emerged as a major mediator of microbiota-derived effects [170, 171]. The microbiota and its metabolites may modulate gastrointestinal and systemic RAS, and RAS alterations may modify microbiota composition and metabolism. In animal models, treatment with microbiota metabolites such as trimethylamine-oxide results in altered expression of RAS receptors in the heart and kidney [170, 172], ACE inhibitory peptides are produced during the bacterial fermentation processes [173], and chronic losartan treatment reduces gut dysbiosis [174]. Consistent with this, the microbiome, acting via RAS regulation, has been related to diabetic-induced kidney injury, hypertension, and organ damage related to hypertension [171, 175]. Interestingly, gut microbiota, via RAS, has been involved in obesity and the development of MetS [176]. As described in a previous section, MetS is related to a higher risk of PD [202, 203], and we have recently shown several potential mechanisms connecting MetS and PD [81, 165].

Given the above-mentioned major role of RAS dysfunction in the inflammatory changes involved in COVID-19, a role of the microbiota/RAS interaction in the pathogenesis and progression of COVID-19 has been suggested [231, 232]. The RAS is suggested to play a pivotal role in inflammatory processes that affect microbiome dysregulation, COVID-19 and the development of PD [233]. As a consequence, prebiotics and probiotics have been suggested for the treatment of RAS-related diseases. Recent studies have shown that probiotics can activate the ACE2/MAS receptor axis [234], and that oral delivery of Ang(1–7)-expressing *Lactobacillus* *paracasei* led to significant modification of microbiota and decreased expression of neuroinflammatory genes in the cortex [235]. However, the microbiome-related effects require further clarification in future studies.

### RAS-related compensatory mechanisms protecting dopaminergic neuron. The intraneuronal RAS

**Fig. 3:**
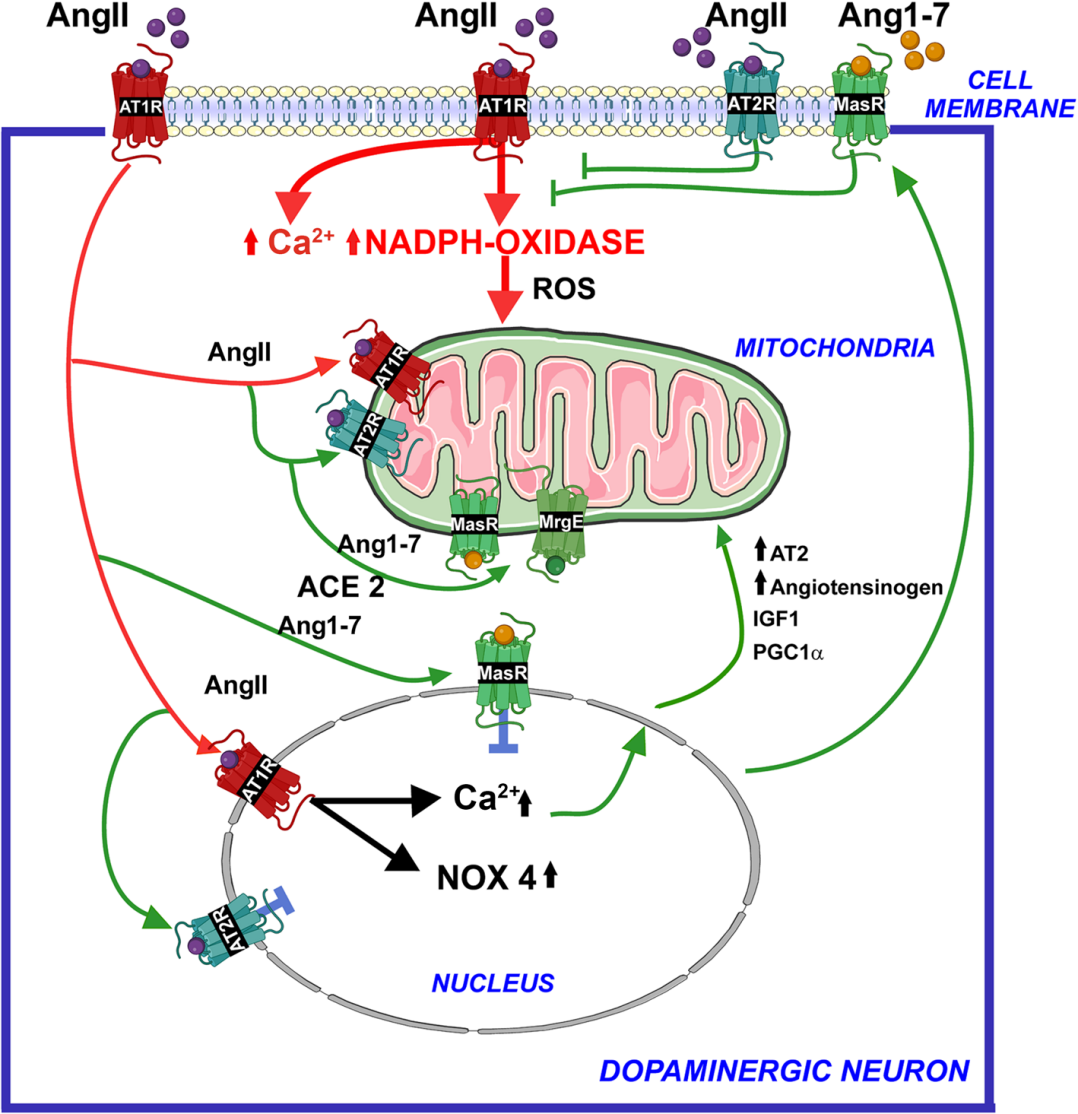
The intraneuronal RAS compensates (green lines: neuroprotective mechanisms) for the pro-oxidative effects of plasma membrane AT1 receptor activation by paracrine AngII (red lines: pro-neurodegenerative mechanisms). Internalization of the AT1/Ang II complex to the nucleus and activation of nuclear and mitochondrial receptors by intracellular AngII and Ang 1–7, trigger several mechanisms that protect neurons against AT1-induced oxidative stress during normal cell function. Antioxidative AT2, Mas, and MrgE receptors are more abundant in the mitochondria. In the nucleus, activation of AT1 receptors triggers several compensatory mechanisms, including increased mRNA expression of antioxidative RAS receptors, angiotensinogen, IGF1, and PGC1α. However, an excess of cell membrane AngII/AT1 receptor activity to compensate for dopamine decrease or other pathogenic factors may overwhelm the buffering mechanisms, leading to the progression of dopaminergic degeneration. AngII, angiotensin II; Ang1-7, angiotensin 1–7; AT1, angiotensin type 1; AT2, angiotensin type 2; MAS, Mas receptors; MrgE, Mas-related receptor MrgE; Nox4, NADPH-oxidase 4; ROS, reactive oxygen species

It has been assumed that peptides of the antioxidative/anti-inflammatory RAS, acting on their corresponding cell surface receptors (AT2, Mas, and Mas-related receptors), counteract the effects of the pro-oxidative AngII/AT1 receptor axis (Fig. 3). However, we have observed that at least in dopaminergic neurons, the intracellular RAS plays a major role in cell protection (Fig. 3). Several studies in peripheral cells have suggested that the intracellular RAS may increase the effects of the pro-oxidative AngII/AT1 receptor axis of the paracrine or tissue RAS [236, 237]. As in several peripheral cells, different RAS receptors are observed in intracellular components of dopaminergic neurons, such as mitochondria and neuronal nuclei [35, 56–58]. However, our studies showed that the nuclear and the mitochondrial RAS constitute a protective mechanism to buffer or counteract excessive pro-oxidative effects of the cell membrane AngII/AT1/NADPH-oxidase (Nox2) effects (Fig. 3), as we reviewed in detail in [38].

In the mitochondria (Fig. 3), different angiotensin receptors modulate oxidative phosphorylation. AT1 receptors, by activating mitochondrial NADPH-oxidase 4 (Nox4), contribute to superoxide production and increase respiration. However, the receptors of the antioxidative system are much more abundant than the AT1 receptors in the mitochondria. We initially observed AT2 and Mas receptors, which induce, via nitric oxide, a downregulation in mitochondrial respiration and modulate oxidative phosphorylation [56, 57]. Interestingly, we observed high levels of mitochondrial ACE2 and its product Ang1-7, which may act on mitochondrial Mas receptors. However, we surprisingly found high mitochondrial levels of the Mas-related receptor E (MrgE), which appear as the most abundant RAS receptor in the mitochondria of dopaminergic neurons [35]. In peripheral cells, ROS-mediated crosstalk between the cell membrane Nox2 and mitochondria has been shown, during which superoxide (and superoxide-derived ROS) produced by Nox2 induces mitoKATP (mitochondrial ATP-sensitive potassium channel) opening, increasing the generation of mitochondrial ROS [127, 139, 238]. We have shown this mechanism also in dopaminergic neurons treated with AngII [128, 129]. This mechanism, triggered by the activation of plasma membrane AT1 receptors, is counteracted by the mitochondrial AT2, Mas, and, particularly, MrgE receptors (Fig. 3).

As described above, activation of AT1 receptors at the cell membrane activates the membrane Nox2, which produces intracellular superoxide that may lead to oxidative stress. However, it is known that activation of AT1 receptors induces a simultaneous internalization of the AngII/AT1 receptor complex towards the cell nucleus, where AngII activates nuclear AT1 receptors, inducing upregulation of nuclear superoxide/H_2_O_2_ (by activating nuclear Nox4) and Ca^2+^ levels (by activating nuclear IP3). This results in regulation of gene expression to trigger several compensatory mechanisms that protect cells against oxidative stress induced by the activation of surface membrane AT1/Nox2 [38, 58] (Fig. 3). These protective mechanisms include (i) increased production of protective AT2 and Mas receptors that traffic to the cell membrane and, particularly, to the mitochondria, (ii) increased production of intracellular angiotensin, particularly Ang1-7 that can act on mitochondrial AT2 and, particularly, MrgE and Mas receptors, respectively, and (iii) upregulation of mRNA expression of cell protective components such as IGF-1 and PGC-1α [38, 58].

In summary, the intracellular RAS may compensate for the deleterious effects of cell membrane AT1 receptor activation. Internalization of the AngII/AT1 complex activates nuclear AT1 receptors, which triggers protective mechanisms against cell membrane AT1-induced oxidative stress. This is possibly effective within physiological levels of AT1 receptor activation. However, excessive AngII/AT1 receptor activation at the cell surface membrane or an increased membrane AT1/AT2-Mas receptor ratio, under RAS deregulatory conditions as those described in the previous sections, may overwhelm the buffering mechanisms, leading to cell oxidative stress and progression of the disease. Interestingly, aging leads to the downregulation of mitochondrial AT2 and Mas/MrgE receptors [35, 56, 57].

In addition to the specific RAS compensatory mechanisms, other neuronal antioxidant systems and protective mechanisms against intracellular calcium dysregulation also protect neurons from the paracrine AngII/AT1 receptor overactivation. Consistent with this, we have shown that AngII also activates the nuclear factor erythroid 2-related factor 2 (NRF2) signaling pathway in dopaminergic neurons [239], which is a key regulator of cell antioxidant mechanisms and redox homeostasis.

### Perspectives, limitations and conclusions

Dopamine/RAS interactions have been observed in several tissues, and dysregulation of these interactions leads to renal, cardiovascular, and other peripheral diseases. In the nigrostriatal system, local RAS dysregulation is involved in major processes responsible for the initiation and progression of dopaminergic neuron degeneration and PD, including oxidative stress, neuroinflammation, and α-synuclein aggregation and transmission. Consistent with this, an imbalance in RAS activity towards the pro-oxidative/pro-inflammatory RAS axis has been observed in the SN and striatum of models exposed to factors associated with dopaminergic degeneration, including aging, menopause, chronic brain hypoperfusion, MetS, gut inflammation, and microbiome dysregulation. Autoantibodies against ACE2 and AT1 receptors are increased in PD models and PD patients and contribute to BBB dysregulation and pro-inflammatory RAS enhancement. Circulating EVs with dysregulated RAS cargo, as observed in MetS models, may also promote neuroinflammation and dopaminergic degeneration. Although the lack of more detailed knowledge of all mechanisms involved in the development and progression of PD limits the development of neuroprotective therapies, current data on the effects of RAS dysregulation, as summarized here, suggest that regulating the brain RAS may be an effective neuroprotective strategy for individuals with a high risk of developing PD or in prodromal stages of PD.

## Data Availability

Not applicable.

## Abbreviations

6-OHDA: 6-Hydroxy dopamine
ACE or ACE1: Angiotensin-converting enzyme
ACE2: Angiotensin-converting enzyme 2
AngII: Angiotensin II
ARBs: AT1 receptor blockers
AT1: Angiotensin type 1
AT1R: AT1 receptors
AT2: Angiotensin type 2
BBB: Blood–brain barrier
CSF: Cerebrospinal fluid
DAG: Diacylglycerol
EVs: Extracellular vesicles
GPCR: G-protein coupled receptors.
IP3: Inositol trisphosphate
MasR: Mas receptors
MetS: Metabolic syndrome
MrgE: Mas-related receptor E
Nox2: NADPH-oxidase
Nox4: NADPH-oxidase 4
PD: Parkinson's disease
RAS: Renin-angiotensin system
ROS: Reactive oxygen species
SN: Substantia nigra
SNpc: Substantia nigra pars compacta
